# 3,4-Dihydroxy­benzaldehyde 4-phenyl­thio­semicarbazone

**DOI:** 10.1107/S1600536808013287

**Published:** 2008-05-10

**Authors:** Kong Wai Tan, Yang Farina, Chew Hee Ng, Mohd Jamil Maah, Seik Weng Ng

**Affiliations:** a Department of Chemistry, University of Malaya, 50603 Kuala Lumpur, Malaysia; b School of Chemical Science and Food Technology, Universiti Kebangsaan Malaysia, 43600 Bangi, Malaysia; c Faculty of Engineering and Science, Universiti Tunku Abdul Rahman, 53300 Kuala Lumpur, Malaysia; d Department of Chemistry, University of Malaya, 50603 Kuala Lumpur, Malaysia

## Abstract

Mol­ecules of the title compound, C_14_H_13_N_3_O_2_S, are linked by inter­molecular O—H⋯O hydrogen bonds into centrosymmetric dimers forming *R*_2_^2^(4) rings which are further linked by O—H⋯S hydrogen bonds and weaker N—H⋯S and N—H⋯O hydrogen bonds to form a three-dimensional network.

## Related literature

For the structure of 2,3-dihydroxy­benzaldehyde thio­semi­carbazone hemihydrate, see: Swesi *et al.* (2006 ▶). For metal derivatives of the title compound, see: Zhu *et al.* (1997 ▶). The graph-set notation is given by Bernstein *et al.* (1995 ▶).
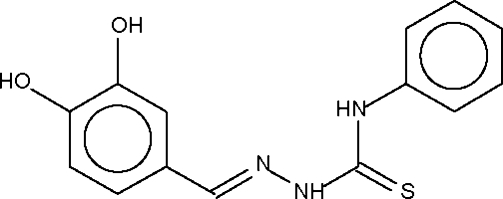


## Experimental

### 

#### Crystal data

C_14_H_13_N_3_O_2_S
*M* *_r_* = 287.33
Monoclinic, 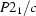

*a* = 9.7261 (2) Å
*b* = 13.1863 (3) Å
*c* = 10.7732 (3) Å
β = 99.055 (2)°
*V* = 1364.46 (6) Å^3^
*Z* = 4
Mo *K*α radiation
μ = 0.24 mm^−1^
*T* = 100 (2) K
0.40 × 0.30 × 0.20 mm

#### Data collection

Bruker SMART APEX diffractometer
Absorption correction: multi-scan (*SADABS*; Sheldrick, 1996 ▶) *T*_min_ = 0.909, *T*_max_ = 0.953
16724 measured reflections
3132 independent reflections
2358 reflections with *I* > 2σ(*I*)
*R*_int_ = 0.078

#### Refinement

*R*[*F* ^2^ > 2σ(*F* ^2^)] = 0.044
*wR*(*F* ^2^) = 0.115
*S* = 1.04
3132 reflections
197 parameters
4 restraints
H atoms treated by a mixture of independent and constrained refinement
Δρ_max_ = 0.40 e Å^−3^
Δρ_min_ = −0.32 e Å^−3^

### 

Data collection: *APEX2* (Bruker, 2007 ▶); cell refinement: *SAINT* (Bruker, 2007 ▶); data reduction: *SAINT*; program(s) used to solve structure: *SHELXS97* (Sheldrick, 2008 ▶); program(s) used to refine structure: *SHELXL97* (Sheldrick, 2008 ▶); molecular graphics: *X-SEED* (Barbour, 2001 ▶); software used to prepare material for publication: *publCIF* (Westrip, 2008 ▶).

## Supplementary Material

Crystal structure: contains datablocks I, global. DOI: 10.1107/S1600536808013287/lh2625sup1.cif

Structure factors: contains datablocks I. DOI: 10.1107/S1600536808013287/lh2625Isup2.hkl

Additional supplementary materials:  crystallographic information; 3D view; checkCIF report

## Figures and Tables

**Table 1:** Hydrogen-bond geometry (Å, °)

*D*—H⋯*A*	*D*—H	H⋯*A*	*D*⋯*A*	*D*—H⋯*A*
O1—H1*o*⋯O2^i^	0.85 (1)	2.03 (2)	2.737 (2)	141 (2)
O2—H2*o*⋯S1^ii^	0.85 (1)	2.34 (1)	3.134 (1)	156 (2)
N2—H2*n*⋯S1^iii^	0.85 (1)	2.73 (1)	3.487 (2)	150 (2)
N2—H2*n*⋯O1^iv^	0.85 (1)	2.56 (2)	3.022 (2)	115 (2)

Symmetry codes: (i) 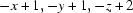; (ii) 
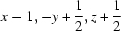
; (iii) 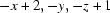; (iv) 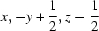.

**Fig. 1.**
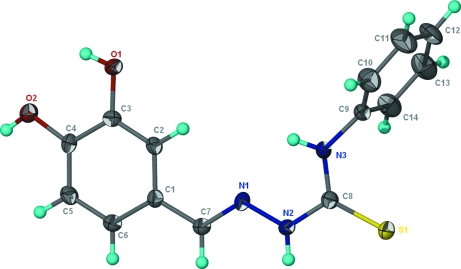
Thermal ellipsoid (Barbour, 2001) plot of C14H13N3O2S at the 70% probability level. Hydrogen atoms are drawn as spheres of arbitrary radii.

## supplementary crystallographic information

### Comment

A previous study of the Schiff bases derived by condensing substituted benzaldehydes with 4-phenylthiosemicarbazides reported the 2,3-dihydroxy compound, which crystallizes as a hemihydrate. The compound features extensive hydrogen bond (Swesi *et al.*, 2006). In the title 3,4-dihydroxy isomer the 4-hydroxy group functions as hydrogen-bond donor to the 3-hydroxy group of a symmetry-related molecule forming R_2_^2^(4) rings (Bernstein *et al.*,  1995). In addition, the 3-hydroxy group is a donor to the sulfur atom of another molecule; the hydrogen bonding arrangement furnishes a three-dimensional network motif. The amino groups are involved in weaker hydrogen bond interactions.

Further work will investigate the formation of metal deratives of the ligand; some metal complexes have been reported by others but these have not characterized by crystallography yet (Zhu *et al.*, 1997).

### Experimental

4-Phenylthiosemicarbazide (0.17 g, 1 mmol) and 3,4-dihydroxybenzaldehyde (0.14 g, 1 mmol) were heated in ethanol (20 ml) for 3 h. Slow evaporation of the solvent yielded yellow crystals.

### Refinement

Carbon-bound H-atoms were placed in calculated positions (C—H 0.95 Å) and were included in the refinement in the riding model approximation, with *U*_iso_(H) set to 1.2 *U*_eq_(C). The hydroxy and amino H-atoms were located in a difference Fourier map, and were refined with a distance retraint of O–H = N–H = 0.85±0.01 Å; their temperature factors were similarly tied.

### Crystal data

**Table:**

C_14_H_13_N_3_O_2_S	*F*_000_ = 600
*M**_r_* = 287.33	*D*_x_ = 1.399 Mg m^−^^3^
Monoclinic, *P*2_1_/*c*	Mo *K*α radiation λ = 0.71073 Å
Hall symbol: -P 2ybc	Cell parameters from 2291 reflections
*a* = 9.7261 (2) Å	θ = 2.5–23.4º
*b* = 13.1863 (3) Å	µ = 0.24 mm^−^^1^
*c* = 10.7732 (3) Å	*T* = 100 (2) K
β = 99.055 (2)º	Block, yellow
*V* = 1364.46 (6) Å^3^	0.40 × 0.30 × 0.20 mm
*Z* = 4	

### Data collection

**Table:**

Bruker SMART APEX diffractometer	3132 independent reflections
Radiation source: fine-focus sealed tube	2358 reflections with *I* > 2σ(*I*)
Monochromator: graphite	*R*_int_ = 0.078
*T* = 100(2) K	θ_max_ = 27.5º
ω scans	θ_min_ = 2.1º
Absorption correction: Multi-scan(SADABS; Sheldrick, 1996)	*h* = −12→12
*T*_min_ = 0.910, *T*_max_ = 0.953	*k* = −17→16
16724 measured reflections	*l* = −13→13

### Refinement

**Table:**

Refinement on *F*^2^	Secondary atom site location: difference Fourier map
Least-squares matrix: full	Hydrogen site location: inferred from neighbouring sites
*R*[*F*^2^ > 2σ(*F*^2^)] = 0.044	H atoms treated by a mixture of independent and constrained refinement
*wR*(*F*^2^) = 0.115	*w* = 1/[σ^2^(*F*_o_^2^) + (0.0502*P*)^2^ + 0.2883*P*] where *P* = (*F*_o_^2^ + 2*F*_c_^2^)/3
*S* = 1.04	(Δ/σ)_max_ = 0.001
3132 reflections	Δρ_max_ = 0.40 e Å^−^^3^
197 parameters	Δρ_min_ = −0.32 e Å^−^^3^
4 restraints	Extinction correction: none
Primary atom site location: structure-invariant direct methods	

### Fractional atomic coordinates and isotropic or equivalent isotropic displacement parameters (Å^2^)

**Table:**

	*x*	*y*	*z*	*U*_iso_*/*U*_eq_	
S1	1.08441 (5)	0.12769 (4)	0.40212 (5)	0.02006 (15)	
O1	0.65919 (15)	0.45705 (10)	0.89678 (14)	0.0221 (3)	
O2	0.40955 (14)	0.39195 (11)	0.94986 (14)	0.0231 (3)	
N1	0.83208 (16)	0.18905 (12)	0.63925 (15)	0.0187 (4)	
N2	0.90695 (16)	0.13520 (12)	0.56294 (16)	0.0185 (4)	
N3	1.03542 (19)	0.27810 (13)	0.55853 (18)	0.0258 (4)	
C1	0.64419 (19)	0.20682 (14)	0.75172 (18)	0.0172 (4)	
C2	0.6914 (2)	0.30261 (14)	0.79586 (18)	0.0177 (4)	
H2	0.7798	0.3262	0.7814	0.021*	
C3	0.61093 (19)	0.36257 (14)	0.85974 (18)	0.0168 (4)	
C4	0.48283 (19)	0.32692 (15)	0.88517 (18)	0.0175 (4)	
C5	0.4360 (2)	0.23207 (15)	0.84415 (19)	0.0207 (4)	
H5	0.3492	0.2077	0.8620	0.025*	
C6	0.5160 (2)	0.17194 (15)	0.77643 (19)	0.0204 (4)	
H6	0.4831	0.1071	0.7471	0.024*	
C7	0.72652 (19)	0.14733 (15)	0.67561 (19)	0.0190 (4)	
H7	0.7022	0.0792	0.6536	0.023*	
C8	1.00702 (19)	0.18502 (14)	0.51400 (19)	0.0177 (4)	
C9	1.1227 (2)	0.35150 (15)	0.5122 (2)	0.0212 (4)	
C10	1.2393 (2)	0.38550 (18)	0.5909 (2)	0.0304 (5)	
H10	1.2651	0.3565	0.6719	0.037*	
C11	1.3183 (3)	0.4624 (2)	0.5504 (2)	0.0386 (6)	
H11	1.3986	0.4863	0.6042	0.046*	
C12	1.2821 (2)	0.50451 (18)	0.4336 (2)	0.0339 (6)	
H12	1.3366	0.5576	0.4070	0.041*	
C13	1.1657 (2)	0.46947 (18)	0.3545 (2)	0.0348 (6)	
H13	1.1410	0.4977	0.2729	0.042*	
C14	1.0858 (2)	0.39327 (17)	0.3946 (2)	0.0287 (5)	
H14	1.0053	0.3696	0.3410	0.034*	
H1O	0.602 (2)	0.4875 (17)	0.935 (2)	0.035 (7)*	
H2O	0.3276 (14)	0.3698 (18)	0.950 (3)	0.043 (8)*	
H2N	0.890 (2)	0.0737 (9)	0.542 (2)	0.036 (7)*	
H3N	0.995 (2)	0.2960 (17)	0.6187 (16)	0.027 (6)*	

### Atomic displacement parameters (Å^2^)

**Table:**

	*U*^11^	*U*^22^	*U*^33^	*U*^12^	*U*^13^	*U*^23^
S1	0.0175 (2)	0.0195 (3)	0.0249 (3)	0.00003 (19)	0.00859 (19)	−0.0035 (2)
O1	0.0209 (7)	0.0162 (7)	0.0316 (9)	−0.0015 (6)	0.0116 (6)	−0.0063 (6)
O2	0.0158 (7)	0.0226 (8)	0.0328 (9)	−0.0011 (6)	0.0093 (6)	−0.0075 (6)
N1	0.0181 (8)	0.0188 (9)	0.0207 (9)	0.0021 (7)	0.0081 (7)	−0.0017 (7)
N2	0.0173 (8)	0.0148 (9)	0.0250 (9)	−0.0013 (7)	0.0086 (7)	−0.0043 (7)
N3	0.0313 (10)	0.0202 (9)	0.0310 (11)	−0.0081 (8)	0.0202 (8)	−0.0076 (8)
C1	0.0159 (9)	0.0187 (10)	0.0178 (10)	−0.0003 (7)	0.0048 (8)	−0.0011 (8)
C2	0.0146 (9)	0.0178 (10)	0.0216 (10)	−0.0011 (7)	0.0056 (8)	−0.0001 (8)
C3	0.0169 (9)	0.0148 (10)	0.0184 (10)	−0.0004 (7)	0.0016 (7)	0.0000 (7)
C4	0.0148 (9)	0.0204 (10)	0.0183 (10)	0.0026 (7)	0.0052 (7)	−0.0008 (8)
C5	0.0141 (9)	0.0206 (10)	0.0285 (12)	−0.0020 (8)	0.0064 (8)	−0.0007 (8)
C6	0.0199 (10)	0.0175 (10)	0.0246 (11)	−0.0030 (8)	0.0061 (8)	−0.0026 (8)
C7	0.0185 (10)	0.0168 (10)	0.0223 (11)	−0.0018 (8)	0.0054 (8)	−0.0027 (8)
C8	0.0145 (9)	0.0167 (10)	0.0224 (10)	0.0011 (7)	0.0046 (8)	−0.0002 (8)
C9	0.0204 (10)	0.0170 (10)	0.0287 (12)	−0.0037 (8)	0.0114 (8)	−0.0041 (8)
C10	0.0317 (12)	0.0345 (13)	0.0248 (12)	−0.0058 (10)	0.0034 (9)	0.0012 (10)
C11	0.0328 (13)	0.0442 (15)	0.0381 (15)	−0.0193 (11)	0.0032 (11)	−0.0047 (11)
C12	0.0323 (13)	0.0250 (12)	0.0474 (16)	−0.0093 (10)	0.0155 (11)	0.0037 (11)
C13	0.0336 (13)	0.0312 (13)	0.0396 (15)	0.0016 (10)	0.0059 (11)	0.0148 (11)
C14	0.0211 (10)	0.0310 (12)	0.0328 (13)	−0.0033 (9)	0.0011 (9)	0.0030 (10)

### Geometric parameters (Å, °)

**Table:**

S1—C8	1.696 (2)	C3—C4	1.398 (3)
O1—C3	1.368 (2)	C4—C5	1.380 (3)
O1—H1O	0.85 (1)	C5—C6	1.395 (3)
O2—C4	1.373 (2)	C5—H5	0.9500
O2—H2O	0.85 (1)	C6—H6	0.9500
N1—C7	1.279 (2)	C7—H7	0.9500
N1—N2	1.378 (2)	C9—C14	1.376 (3)
N2—C8	1.348 (2)	C9—C10	1.380 (3)
N2—H2N	0.85 (1)	C10—C11	1.383 (3)
N3—C8	1.331 (3)	C10—H10	0.9500
N3—C9	1.428 (3)	C11—C12	1.370 (3)
N3—H3N	0.84 (1)	C11—H11	0.9500
C1—C6	1.393 (3)	C12—C13	1.385 (3)
C1—C2	1.401 (3)	C12—H12	0.9500
C1—C7	1.461 (3)	C13—C14	1.381 (3)
C2—C3	1.372 (3)	C13—H13	0.9500
C2—H2	0.9500	C14—H14	0.9500
			
C3—O1—H1O	110.7 (17)	C5—C6—H6	119.9
C4—O2—H2O	110.6 (18)	N1—C7—C1	118.55 (17)
C7—N1—N2	119.07 (16)	N1—C7—H7	120.7
C8—N2—N1	117.64 (16)	C1—C7—H7	120.7
C8—N2—H2N	119.3 (17)	N3—C8—N2	115.47 (17)
N1—N2—H2N	123.0 (17)	N3—C8—S1	125.24 (15)
C8—N3—C9	126.89 (17)	N2—C8—S1	119.28 (15)
C8—N3—H3N	116.2 (16)	C14—C9—C10	120.35 (19)
C9—N3—H3N	116.9 (16)	C14—C9—N3	120.67 (19)
C6—C1—C2	119.21 (17)	C10—C9—N3	118.8 (2)
C6—C1—C7	121.05 (18)	C9—C10—C11	119.2 (2)
C2—C1—C7	119.68 (17)	C9—C10—H10	120.4
C3—C2—C1	120.50 (17)	C11—C10—H10	120.4
C3—C2—H2	119.7	C12—C11—C10	120.8 (2)
C1—C2—H2	119.7	C12—C11—H11	119.6
O1—C3—C2	118.33 (17)	C10—C11—H11	119.6
O1—C3—C4	121.62 (17)	C11—C12—C13	119.8 (2)
C2—C3—C4	120.06 (17)	C11—C12—H12	120.1
O2—C4—C5	123.84 (17)	C13—C12—H12	120.1
O2—C4—C3	116.05 (17)	C14—C13—C12	119.7 (2)
C5—C4—C3	120.10 (18)	C14—C13—H13	120.1
C4—C5—C6	119.95 (18)	C12—C13—H13	120.1
C4—C5—H5	120.0	C9—C14—C13	120.1 (2)
C6—C5—H5	120.0	C9—C14—H14	119.9
C1—C6—C5	120.14 (18)	C13—C14—H14	119.9
C1—C6—H6	119.9		
			
C7—N1—N2—C8	172.24 (18)	C2—C1—C7—N1	−7.8 (3)
C6—C1—C2—C3	−1.7 (3)	C9—N3—C8—N2	−171.35 (19)
C7—C1—C2—C3	175.33 (18)	C9—N3—C8—S1	7.8 (3)
C1—C2—C3—O1	−177.55 (17)	N1—N2—C8—N3	8.7 (3)
C1—C2—C3—C4	2.2 (3)	N1—N2—C8—S1	−170.51 (13)
O1—C3—C4—O2	−0.4 (3)	C8—N3—C9—C14	66.2 (3)
C2—C3—C4—O2	179.82 (17)	C8—N3—C9—C10	−118.6 (2)
O1—C3—C4—C5	178.64 (18)	C14—C9—C10—C11	0.3 (3)
C2—C3—C4—C5	−1.1 (3)	N3—C9—C10—C11	−174.9 (2)
O2—C4—C5—C6	178.51 (18)	C9—C10—C11—C12	−0.2 (4)
C3—C4—C5—C6	−0.5 (3)	C10—C11—C12—C13	−0.5 (4)
C2—C1—C6—C5	0.1 (3)	C11—C12—C13—C14	1.0 (4)
C7—C1—C6—C5	−176.89 (18)	C10—C9—C14—C13	0.1 (3)
C4—C5—C6—C1	1.0 (3)	N3—C9—C14—C13	175.3 (2)
N2—N1—C7—C1	−177.22 (16)	C12—C13—C14—C9	−0.8 (4)
C6—C1—C7—N1	169.23 (19)		

### Hydrogen-bond geometry (Å, °)

**Table:**

*D*—H···*A*	*D*—H	H···*A*	*D*···*A*	*D*—H···*A*
O1—H1O···O2^i^	0.85 (1)	2.03 (2)	2.737 (2)	141 (2)
O2—H2O···S1^ii^	0.85 (1)	2.34 (1)	3.134 (1)	156 (2)
N2—H2N···S1^iii^	0.85 (1)	2.73 (1)	3.487 (2)	150 (2)
N2—H2N···O1^iv^	0.85 (1)	2.56 (2)	3.022 (2)	115 (2)

Symmetry codes: (i) −*x*+1, −*y*+1, −*z*+2; (ii) *x*−1, −*y*+1/2, *z*+1/2; (iii) −*x*+2, −*y*, −*z*+1; (iv) *x*, −*y*+1/2, *z*−1/2.
